# IgE is associated with exacerbations and lung function decline in COPD

**DOI:** 10.1186/s12931-021-01847-0

**Published:** 2022-01-04

**Authors:** Marek Lommatzsch, Timotheus Speer, Christian Herr, Rudolf A. Jörres, Henrik Watz, Achim Müller, Tobias Welte, Claus F. Vogelmeier, Robert Bals, for the COSYCONET study group

**Affiliations:** 1 University of Rostock, Rostock, Germany; 2 Department of Internal Medicine IV, Nephrology, Saarland University Hospital, Homburg, Germany; 3 Department of Internal Medicine V - Pulmonology, Allergology, Critical Care Medicine, Saarland University Medical Center, 66421 Homburg, Germany; 4 University of Munich, Munich, Germany; 5 Airway Research Center North (ARCN), German Center for Lung Research (DZL), Pulmonary Research Institute at LungenClinic Grosshansdorf, Grosshansdorf, Germany; 6 Boehringer Ingelheim Pharma GmbH & Co. KG, Ingelheim, Germany; 7 German Center for Lung Research (DZL), University of Hannover, Hannover, Germany; 8 German Center for Lung Research (DZL), Department of Medicine, Pulmonary and Critical Care Medicine, University Medical Center Giessen and Marburg, Philipps-Universität Marburg, Marburg, Germany

**Keywords:** COPD, IgE, Exacerbations, Lung function decline

## Abstract

**Background:**

Both allergen-specific IgE and total IgE in serum play a major role in asthma. However, the role of IgE in chronic obstructive pulmonary disease (COPD) is poorly understood. It was the aim of this study to systematically analyze the relationship between serum IgE levels and disease characteristics in large COPD cohorts.

**Methods:**

COSYCONET is a comprehensively characterized cohort of patients with COPD: total IgE and IgE specific to common aeroallergens were measured in serum of 2280 patients, and related to clinical characteristics of the patients. WISDOM is another large COPD population (2477 patients): this database contains the information whether total IgE in serum was elevated (≥ 100 IU/l) or normal in patients with COPD.

**Results:**

Both in COSYCONET and WISDOM, total IgE was elevated (≥ 100 IU/l) in > 30% of the patients, higher in men than in women, and higher in currently than in not currently smoking men. In COSYCONET, total IgE was elevated in patients with a history of asthma and/or allergies. Men with at least one exacerbation in the last 12 months (50.6% of all men in COSYCONET) had higher median total IgE (71.3 IU/l) than men without exacerbations (48.3 IU/l): this difference was also observed in the subgroups of not currently smoking men and of men without a history of asthma. Surprisingly, a history of exacerbations did not impact on total IgE in women with COPD. Patients in the highest tertiles of total IgE (> 91.5 IU/ml, adjusted OR: 1.62, 95% CI 1.12–2.34) or allergen-specific IgE (> 0.19 IU/ml, adjusted OR: 2.15, 95% CI 1.32–3.51) were at risk of lung function decline (adjusted by: age, gender, body mass index, initial lung function, smoking status, history of asthma, history of allergy).

**Conclusion:**

These data suggest that IgE may play a role in specific COPD subgroups. Clinical trials using antibodies targeting the IgE pathway (such as omalizumab), especially in men with recurrent exacerbations and elevated serum IgE, could elucidate potential therapeutic implications of our observations.

**Supplementary Information:**

The online version contains supplementary material available at 10.1186/s12931-021-01847-0.

## Introduction

Allergen-specific Immunoglobulin E (IgE) is postulated to be a key driver of exacerbations in allergic asthma [1]. Clinical trials supported this concept and led to the approval of the anti-IgE antibody omalizumab for the treatment of severe allergic asthma [2, 3]. In addition, several studies discovered a genuine role for total IgE in asthma, independent from allergen-specific IgE [4, 5]. Indeed, anti-IgE treatment was also effective in patients without evidence of allergies [6]. Of note, anti-IgE treatment enhances antiviral immunity via a downregulation of the high-affinity IgE receptor (FcεRI) on plasmacytoid dendritic cells (pDCs) [7], resulting in a reduction of viral exacerbations in asthma [8, 9]. The strong correlation of the FcεRI expression on pDCs with total IgE in serum [6] supported the idea that total IgE in serum plays a genuine role in asthma, independent from allergen-specific IgE [10].

The finding that FcεRI is not only upregulated on pDCs in asthma, but also in COPD, led to the hypothesis that anti-IgE treatment might also reduce exacerbations in COPD [11]. However, there is still little information on the role and significance of total IgE serum concentrations in COPD. Three small studies investigated the relationship between lung function and total serum IgE in COPD, but yielded conflicting results [12–14]. Larger studies and meta-analyses focussed on allergen-specific IgE and postulated that allergen-specific IgE antibodies might be biomarkers for COPD subtypes with asthma and/or atopy [15–17]. However, there is limited data on the relationship between total IgE concentrations in serum and typical clinical characteristics of patients with COPD, including airflow limitation, lung function decline and the occurrence of exacerbations. It was the aim of this analysis, therefore, to systematically explore this relationship in two large clinical COPD cohorts: COSYCONET and WISDOM [18–20].

## Methods

The multicenter cohort study COSYCONET (German COPD and Systemic Consequences—Comorbidities Network) investigates the phenotypes and progression of COPD and its comorbidities, as described [20]. In 31 study centers in Germany, 2741 patients with physician-diagnosed COPD were recruited between 2010 and 2013 (the study was approved by the ethics committees of the participating centers, all participants provided written informed consent) [20]. At baseline, structured interviews were performed to assess disease characteristics, comorbidities, medication and demographics. The severity of COPD was categorized spirometrically (GOLD grades 0–4) and clinically (GOLD groups A–D) [21]. COSYCONET comprises visits at baseline (visit 1), and after 6, 18, and 36 months (visit 4). Changes in lung function were determined between visit 1 and 4. WISDOM was a randomized trial in which patients with COPD received tiotropium, salmeterol, and fluticasone propionate daily for 6 weeks and were then randomly assigned to receive either continued ICS or bronchodilators only after a stepwise ICS withdrawal [18, 19]. In contrast to COSYCONET, all patients from the WISDOM study had a history of exacerbations and a FEV_1_ < 50% predicted. Data from 2477 patients were available. The WISDOM database contains the information whether patients had elevated (≥ 100 IU/l) or normal (< 100 IU/l) serum IgE levels.

### Measurements of immunoglobulin E (IgE)

Total and allergen-specific (SX-1) IgE concentrations in serum were determined using the ImmunoCAP system (Thermo Fisher Scientific, Uppsala, Sweden). The SX1 screening panel detects 8 allergen-specific IgE antibodies: against house dust mite (Dermatophagoides pteronyssinus), cat, dog, birch, timothy (Phleum pratense), rye, ragweed, Cladosporium herbarum.

### Statistical analysis

Statistical analyses were performed using SPSS (Version 23; IBM Corp., Armonk, NY, USA). IgE in serum was not normally distributed. Therefore, the Mann–Whitney U-test was used to determine differences in IgE between subgroups of the cohort. Correlation analyses were performed using Spearman’s correlation coefficient. Probability values of p < 0.05 were regarded as significant. Box plots display the median (line within the box), interquartile range (edges of the box) and extremes (vertical lines). Restricted cubic splines were used as an unbiased approach to visualize the non-linear association between total or allergen-specific IgE (SX1) and the exacerbation history. Three knots of total or allergen-specific IgE were placed corresponding to the 10th, 50th, and 90th percentile of the IgE levels, respectively. Moreover, to assess the association between total or allergen-specific IgE at baseline and changes of FEV_1_ during follow-up, we performed group-based trajectory modeling of FEV_1_ using the STATA/IC 15 package TRAJ (StrataCorp LLC, Texas, USA). This approach is based on the SAS PROC TRAJ macro kit and fits a semiparametric (discrete mixture) model for longitudinal data using the maximum likelihood method. We used the Bayesian information criterion to establish the optimal number of groups with at least 5% of participants in the smallest trajectory. Trajectory groups were termed group A (increasing FEV_1_), group B (stable FEV_1_), and group C (declining FEV_1_). Afterwards, logistic regression analyses were performed to assess the association between baseline IgE levels and trajectory group C (declining FEV_1_) using group B as the reference category. The analysis was adjusted for age, gender, body mass index (BMI), initial FEV_1_ (% predicted), and smoking status.

## Results

### Patient characteristics: COSYCONET and WISDOM

In COSYCONET, total IgE at baseline (visit 1) was available in 2280, allergen-specific IgE (SX1) in 2326 patients. Both parameters were documented in 2263 patients. Table 1 shows characteristics of patients with documented total IgE (2280 patients): 942 females (41.3%), 1338 males (58.7%); 576 current smokers (patients who smoked within the last 4 weeks prior to visit 1: 24.9%), 1527 ex-smokers (67.3%), 177 never smokers (7.8%). There were 1231 patients (54%) with at least one exacerbation in the last 12 months prior to visit 1, and 1048 patients (46%) without an exacerbation (data missing for 1 patient). The majority was in spirometric GOLD grades 2 (41.8%) or 3 (32.4%) (Additional file 1: Table S1). In WISDOM [18, 19], there were 829 current smokers (33.5%), 1648 ex-smokers (66.5%) and 0 never smokers (0%); 82.5% of all patients were males. All patients in WISDOM had a history of exacerbations and a FEV_1_ < 50% predicted (GOLD grades 3 and 4).

**Table 1:** Patient characteristics (COSYCONET)

Parameter (visit 1)	Unit	Median	Interquartile range	Mean	Standard deviation
COSYCONET (n = 2280 patients)					
Age	Years	66.0	59.0–71.0	64.8	8.6
Years since diagnosis	Years	6.0	3.0–10.0	7.6	6.7
Weight	kg	77.0	66.0–90.0	79.0	18.0
Height	Cm	170.0	164.0–177.0	170.5	9.2
BMI	kg/m^2^	26.5	23.4–30.1	27.1	5.4
Pack years	Years	42.0	22.5–64.5	48.4	35.6
CAT score	Points	18.0	13.0–23.0	18.0	7.3
mMRC score	Points	1.0	1.0–2.0	1.6	0.9
6MWT distance	Meters	435.0	360.0–492.8	422.5	107.4
BODE-Index	Points	2.0	1.0–3.0	2.2	2.0
SGRQ	Points	56.0	40.2–71.7	55.2	21.4
No. of Exacerbations	Number	1.0	0–2	1.3	2.8
FEV_1_	Liters	1.5	1.1–2.1	1.7	0.7
FEV_1_ (% predicted)	%	55.0	41.0–72.0	57.0	21.1
FEV1%FVC	%	54.8	44.6–65.5	55.2	13.8
ITGV (% predicted)	%	140.6	117.6–168.2	143.9	37.1
DLCO (% predicted)	%	53.7	40.0–69.6	55.3	21.6
Total IgE	IU/ml	46.2	17.3–139.4	176.8	496.1
Allergen-specific IgE (SX1)	IU/ml	0.11	0.07–0.34	3.04	16.13

BMI, Body mass index; CAT, COPD assessment test; mMRC, Modified Medical Research Council; 6MWT, 6 min walk test; BODE, body-mass index, airflow obstruction, dyspnea, and exercise capacity index; SGRQ, St. George's Respiratory Questionnaire; FEV_1_, Forced expiratory volume in the first second of expiration; ITGV, intra-thoracic gas volume; DLCO, lung diffusion capacity for carbon monoxide

### IgE in serum: COSYCONET and WISDOM

In COSYCONET, allergen-specific IgE (SX1) was elevated (≥ 0.35 IU/ml) in 24.8% of the total cohort (22.8% of all women, 26.3% of all men with COPD). There was a correlation (r = 0.38, p < 0.001) between total and allergen-specific IgE (SX1) in COSYCONET (Additional file 1: Fig. S1). Percentages of patients with elevated total IgE (≥ 100 IU/ml) in COSYCONET and WISDOM are shown in Table 2. Additional file 1: Tables S2A and B compare characteristics of patients with normal or elevated total or allergen-specific IgE (SX1) in COSYCONET.

**Table 2:** Proportion of patients with elevated total IgE in serum

IgE ≥ 100 IU/l in serum	COSYCONET (n = 2280 patients) (%)	WISDOM (n = 2477 patients) (%)
Total population	31.2	34.2
All males	36.6	35.4
Male current smokers	41.1	36.0
Male ex-smokers	34.7	35.0
All females	23.6	28.7
Female current smokers	25.2	30.7
Female ex-smokers	22.7	27.5

Shown are the percentages of patients with elevated total IgE levels in serum (≥ 100 IU/l), in the COSYCONET study (left column) and in the WISDOM study (right column), for the total population and the subgroups according to gender and smoking

### Gender and smoking: COSYCONET and WISDOM

**Fig. 1:**
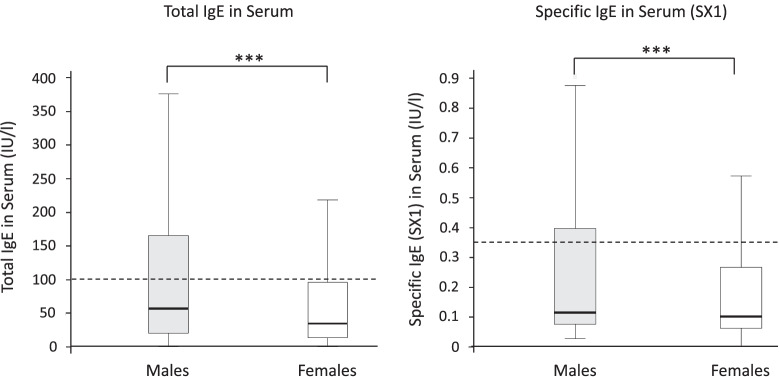
Distribution of IgE in serum. Shown are total IgE and allergen-specific IgE (as measured by the SX1 screening test) levels in the COSYCONET cohort. Box plots display the median (line within the box), interquartile range (edges of the box) and extremes (vertical lines). The dotted lines show currently accepted cut-offs for normal values. The differences between men and women were highly significant (***: p < 0.001)

In COSYCONET, allergen-specific IgE (SX1) was higher in men than in women (Fig. 1). The proportion of patients with elevated total IgE was higher in men than in women, both in COSYCONET and in WISDOM (Table 2). In COSYCONET, total IgE was higher in currently smoking than in not currently smoking men (median: 74.2 IU/ml vs. 53.2 IU/ml, p = 0.02): this difference was not significant in women (median: 38.9 IU/ml vs. 32.5 IU/ml, p = 0.14). In both studies, a higher proportion of patients with elevated total IgE was found in current smokers than in ex-smokers (Table 2). Allergen-specific IgE levels were not affected by current smoking in COSYCONET, neither in women nor in men (data not shown).

### Age, asthma, allergies and eosinophils: COSYCONET

**Fig. 2:**
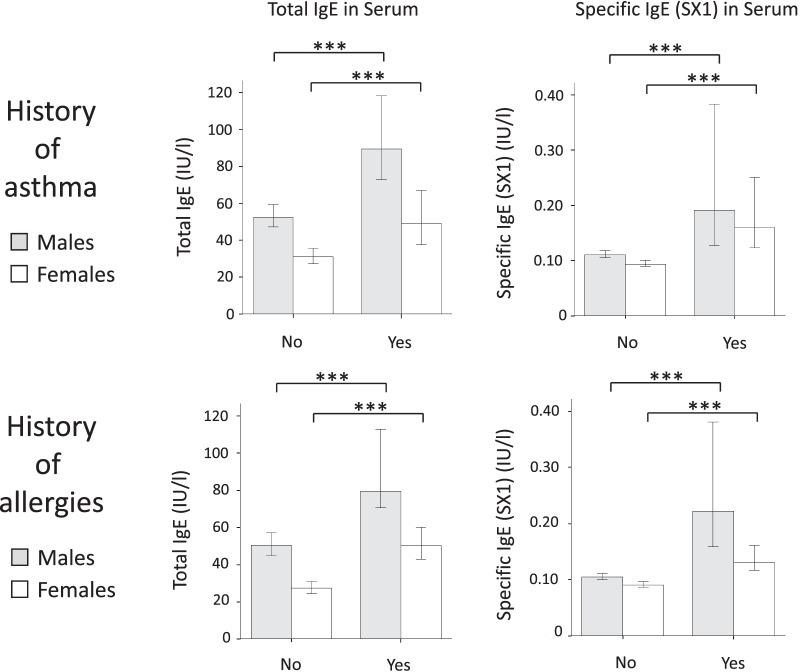
IgE and history of asthma or allergies. Shown are total IgE and allergen-specific IgE (as measured by the SX1 screening test) levels in patients with or without a history of asthma or allergies (COSYCONET cohort). The bars show median values with interquartile ranges. ***: p < 0.001

In COSYCONET, there was no correlation between the age of the participants and total or allergen-specific IgE, neither in the total group nor in the subgroups of men and women with COPD (data not shown). Both in females and males, total IgE and allergen-specific IgE (SX1) levels were higher in patients with a history of asthma (414 patients, 18.2% of the total cohort) or self-reported allergies (765 patients, 33.6% of the total cohort), as compared to patients without a history of asthma or allergies (Fig. 2). Data on blood eosinophil counts were only available in 594 out of 2280 patients (26.1%). Patients with elevated total or allergen-specific IgE had slightly higher blood eosinophils than those with normal IgE levels (Additional file 1: Table S3).

### IgE and lung function decline: COSYCONET

**Fig. 3:**
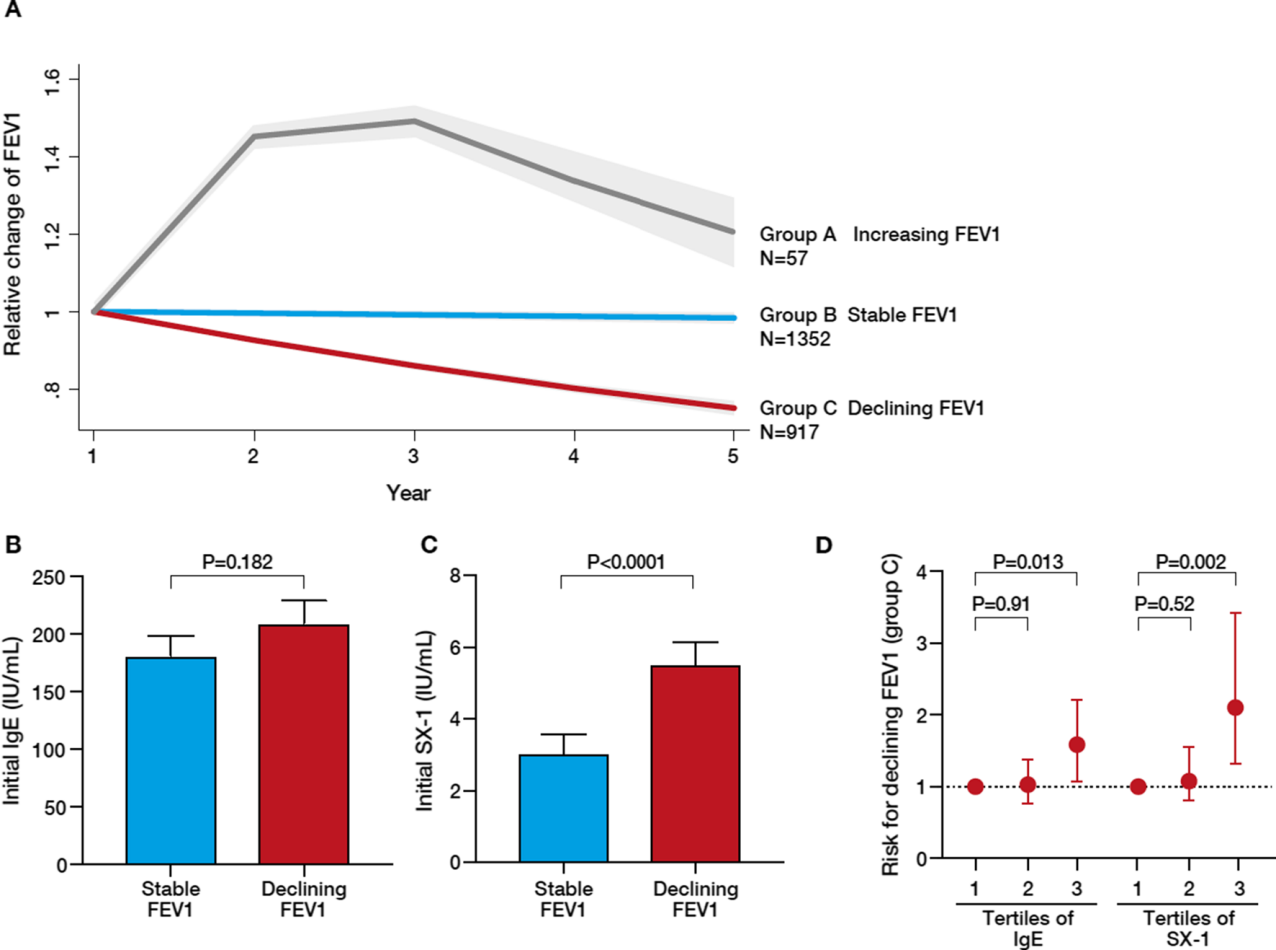
IgE and risk of lung function decline. **A** Lung function trajectories in the COSYCONET cohort. Shown are relative changes in FEV_1_ in the 3 groups (group A: increasing FEV_1_; group B: stable FEV_1_; group C: declining FEV_1_). **B** and **C** Comparison of mean concentrations of total IgE (**B**) or allergen-specific IgE (as measured by the SX1 screening test, **C**) between group B (stable FEV_1_) and group C (declining FEV_1_). **D** Risk of declining FEV_1_, according to the tertiles of total IgE or allergen-specific IgE (SX1) in serum

Total IgE did not correlate with baseline FEV_1_, DLCO or parameters of hyperinflation (RV, ITGV) (data not shown). Total and allergen-specific IgE according to spirometric GOLD grades are detailed in the (Additional file 1: Fig. S2). To describe changes of FEV_1_ during follow-up, group-based trajectory modeling of FEV_1_ was performed. Using this approach, three groups of FEV_1_ changes were identified: group A, increasing FEV_1_; group B, stable FEV_1_; group C, declining FEV_1_ (Fig. 3A). While total IgE levels did not differ (p = 0.182) between group B and C (Fig. 3B), allergen-specific IgE (SX1) levels were higher (p < 0.0001) in group C as compared with group B (Fig. 3C). Logistic regression analyses (adjusted by age, gender, body mass index, initial FEV_1_, smoking status, history of asthma, history of allergy) revealed that the highest tertiles of total IgE (> 91.5 IU/ml) and allergen-specific IgE (> 0.19 IU/ml) were both associated with a significant risk for lung function decline (Fig. 3D and Additional file 1: Tables S4A and B).

### IgE and exacerbation history: COSYCONET

**Fig. 4:**
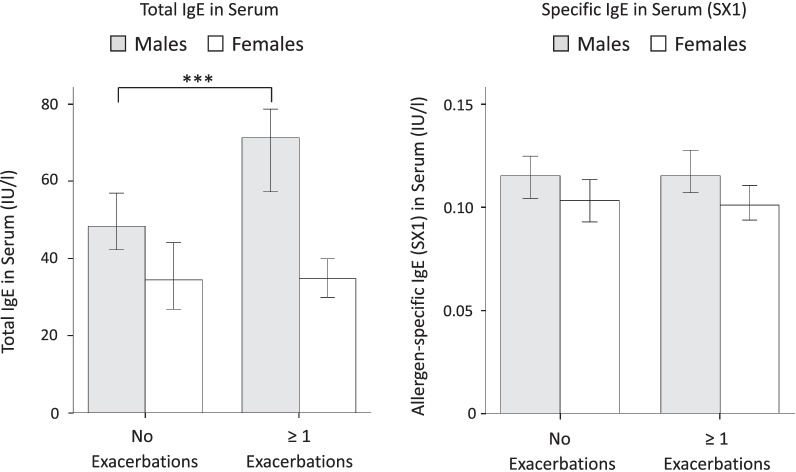
IgE and exacerbation history. Shown are total IgE and allergen-specific IgE (as measured by the SX1 screening test) levels in patients without and in patients with at least one exacerbation in the last 12 months prior to IgE measurement (COSYCONET cohort). The bars show median values with interquartile ranges. The difference between men with and without exacerbations was highly significant (***: p < 0.001)

Men with at least one exacerbation in the last 12 months (50.6% of all men) displayed higher total IgE (median: 71.3 IU/ml) than men without exacerbations (median: 48.3 IU/ml): this difference was not found in women (Fig. 4). Increasing total IgE levels were associated with a higher risk of exacerbations in men (p = 0.004), but not in women (p = 0.135) with COPD (Additional file 1: Fig. S3). Because total IgE levels in men were affected by current smoking or a history of asthma, we tested this association in the subgroups of men who were not currently smoking (1029 men: 76.9% of all men in the cohort) and of men without a history of asthma (1150 men: 85.9% of all men in the cohort). Not currently smoking men with at least one exacerbation displayed higher total IgE (median: 64.0 IU/ml) than those without exacerbations (median: 46.3 IU/ml) (p < 0.01). Men without a history of asthma with at least one exacerbation displayed significantly higher total IgE (median: 59.0 IU/ml) than those without exacerbations (median: 48.2 IU/ml) (p < 0.05). Multivariable analysis revealed a significant association of total IgE with a history of exacerbations in men with COPD (p = 0.02), when adjusted by gender, history of asthma, history of allergies and smoking status: this association was not significant in women with COPD (p = 0.50). There was no difference in allergen-specific IgE between patients with and without exacerbations (Fig. 4).

## Discussion

This study is the first to demonstrate that total IgE is elevated in specific COPD subgroups: in men with exacerbations and in patients with lung function decline. The data suggest that IgE may play a role in specific COPD subgroups.

Elevated total IgE (≥ 100 IU /ml) was found in 31.2% of the patients in COSYCONET. In WISDOM, this proportion was slightly higher (34.2%), most likely due to the higher percentage of males (WISDOM: 82.5%, COSYCONET: 58.7%): percentages of men with elevated IgE were similar (WISDOM: 35.4%, COSYCONET: 36.6%). These data are confirmed by the COPDGene study (899 patients with no history of asthma; 832 with a history of asthma), where 34.3% of the patients had elevated total IgE [17]. The largest study on normal values of serum IgE in Europe was the SAPALDIA survey in Switzerland [22], which consisted of 8344 subjects (49.7% females): 3680 never-smokers, 1888 former, 2776 current smokers. In SAPALDIA, 19.9–27.5% of the subjects (depending on the smoking status) had elevated total IgE. The geometric mean of total IgE in SAPALDIA (31.1 IU/l) was lower than the median total IgE in COSYCONET (46.2 IU/l). In a general population of Norwegian adults (1512 subjects) [23], the geometric means of total IgE were even lower (10–17 IU/l in women, 15.5–20.9 IU/l in men). These data suggest that patients with COPD have higher total IgE levels than the general population. Whether this elevation is explained by the smoking history [24] of the patients is currently unclear.

Both in COSYCONET and WISDOM, there were higher total IgE levels in men than in women, and in currently smoking than in not currently smoking men. This impact of gender and smoking on total IgE is in accordance with the literature. In SAPALDIA [22], total IgE was higher in men (≥ 100 IU/ml: 23.7–30.5%) than in women (≥ 100 IU/ml: 14.8–23.3%), and in currently smoking men (geometric mean: 45.1 IU/l) than in not currently smoking men (geometric mean: 33.9–34.3 IU/l). Similar effects were observed in the Norwegian study [23], and studies from Denmark [25] and UK [26]. More recent studies confirmed that males display higher total and allergen-specific IgE than females [27, 28], and that this difference is already detectable in early childhood [29]. It has been hypothesized that sex hormone effects on the innate and adaptive Immune system account for this difference [30]. The effect of smoking on total IgE appears to be dose-dependent [31]. There are several hypotheses to explain the effects of smoking on IgE levels [32], one postulates a smoking-induced increase in Interleukin-4 (IL-4) secretion by T-cells, leading to increased IgE production [24].

Exacerbations have a major impact on disease progression and treatment strategies in COPD [33–35]. We found that men with exacerbations had higher total IgE than men without exacerbations. Strikingly, this effect was not observed in women. In addition, there was no difference in allergen-specific IgE between these two groups. Although there is a well-established correlation between total and allergen-specific IgE [22, 23], which has also been observed in our study, there is evidence for a distinct role of total IgE in asthma [4]. Several studies supported the hypothesis that total IgE represents a polyclonal mixture of antibodies, which is rather driven by bacterial superantigens, then by aeroallergens [36]. Indeed, anti-IgE treatment reduces exacerbations even in those patients with asthma who do not have any evidence of allergies [6], suggesting that elevated total IgE and the effects of anti-IgE treatment are not primarily related to the presence of allergen-specific IgE. Elevated total IgE in men with COPD exacerbations might, therefore, reflect recurrent local stimulations in the airways with bacterial (super) antigens. In turn, the resulting IgE elevation could increase the FcεRI expression on pDCs [11] and hamper anti-viral immune responses [7], leading to a vicious circle with recurrent exacerbations.

COPD may show a wide variation regarding change of lung function over time [37]. We found that patients with high total IgE and/or high allergen-specific IgE were at an increased risk for lung function decline. It is of note that this risk was (1) independent of the gender and (2) found both for elevated total IgE and allergen-specific IgE. Notably, there was a correlation between allergen-specific IgE (but not total IgE) and the FEV_1_, both in male and in female patients with COPD. These data suggest that allergen-specific IgE might play a hitherto unrecognized role in the pathogenesis of lung function abnormalities in COPD [37]. It needs to be noted that a history of asthma and allergies was not an exclusion criterion in COSYCONET. Indeed, higher levels of total and allergen-specific IgE were found in patients with a history of asthma and/or allergies. However, it appears unlikely that the impact of IgE on lung function decline is solely explained by a history allergies and/or asthma. There is substantial evidence that elevated IgE levels in asymptomatic, nonatopic subjects are associated with worse lung function and an accelerated lung function decline [25, 38–40].

This study has several strengths. First, it analyzes a large number of patients with COPD (COSYCONET: 2280 patients; WISDOM: 2477 patients). Second, the German COSYCONET cohort is a well characterized cohort, with extensive information on the history and the characteristics of the patients. Finally, lung function data in COSYCONET were available over a period of 3 years, enabling an analysis of the relationship between IgE and lung function trajectories. Therefore, the COSYCONET dataset offered a unique opportunity to analyze the relationship between serum IgE and clinical characteristics of patients with COPD. Moreover, we did not only measure total IgE, but also allergen-specific IgE using the SX1 screening test which comprises 8 common aeroallergens (house dust mite, cat, dog, birch, timothy, rye, ragweed, cladosporium herbarum). Thus, we were able to analyze effects of total IgE and allergen-specific IgE separately.

There are several limitations. First, due to the large number of serum samples in the COSYCONET cohort, the analysis of allergen-specific IgE was limited to a screening panel of 8 common aeroallergens. Several relevant allergens might have, therefore, been missed in this analysis [41]. In addition, a separate analysis of the relevance of IgE antibodies against single allergens (such as birch or cat) could not be performed. Second, we did not measure the high-affinity IgE receptor on pDCs in the patients, and could not correlate the pDC high-affinity IgE receptor expression with IgE serum levels or the exacerbation history. Another major limitation was the paucity of blood eosinophil data (only available in 26.1% of the patients at time point 1). Therefore, given the major role of blood eosinophils as a prognostic biomarker for exacerbations in COPD [42] and in asthma [43] and the variation of blood eosinophils over time [44] and in response to changing doses of inhaled medications [45], further studies are needed to come to a firm conclusion on the relationship between blood eosinophils and serum IgE in COPD, especially in patients with recurrent exacerbations. Furthermore, this was a post-hoc analysis of the COSYCONET cohort with the natural limitations of cross-sectional studies. Therefore, prospective studies on the role of IgE in COPD are needed to validate the results of our study. Finally, due to the limitations of the WISDOM study database (only patients with an exacerbation history; only dichotomous information on serum IgE levels; short study period), the findings from the COSYCONET cohort regarding exacerbations and lung function decline could not be validated in the WISDOM study population.

## Conclusions

Elevated serum IgE is associated with the occurrence of exacerbations in men with COPD and with the risk of lung function decline. We speculate that IgE-mediated pathways might be involved in the pathogenesis of exacerbations in men with COPD and in the pathogenesis of progressive airflow limitation in patients with elevated IgE levels. Clinical trials using antibodies targeting the IgE pathway (such as omalizumab), especially in men with recurrent exacerbations and elevated total serum IgE, could elucidate potential therapeutic implications of our observations.

## Supplementary Information

**Additional file 1.** Supplementary Tables and Figures.

## Data Availability

Individual de-identified participant data of the studies can be shared upon reasonable request.

## Abbreviations

COPD: Chronic obstructive pulmonary disease
DLCO: Diffusing capacity for carbon monoxide
FcεRI: High-affinity IgE receptor
FEV_1_: Forced expiratory volume in the first second of expiration
GOLD: Global initiative for chronic obstructive lung disease
IgE: Immunoglobulin E
ITGV: Intrathoracic gas volume
pDC: Plasmacytoid dendritic cell
RV: Residual volume
