# Supplementary material for: IgE is associated with exacerbations and lung function decline in COPD
Source: Respir Res. 2022 Jan 4;23:1. doi: 10.1186/s12931-021-01847-0 (PMC8725269; doi:10.1186/s12931-021-01847-0)
Supplement: Supplementary file 1 — Additional file 1. Supplementary Tables and Figures. [file 12931_2021_1847_MOESM1_ESM.pdf]

IgE is associated with exacerbations and lung function decline in COPD

Marek Lommatzsch [a], Timotheus Speer [b], Christian Herr [c], Rudolf A. Jörres [d], Henrik Watz [e], Achim Müller [f], Tobias Welte [g], Claus F. Vogelmeier [h], and Robert Bals [c§], for the COSYCONET study group

Additional File 1

Table S1

Spirometric grades and clinical groups according to GOLD.

Spirometric grades	No. of patients	Percentage
0/1	388	17,0
2	954	41,8
3	738	32,4
4	193	8,5
Missing data	7	0,3
**Groups**	**No. of patients**	**Percentage**
A	256	11,2
B	1194	52,4
C	37	1,6
D	779	34,2
Missing data	14	0,6

Table S2A

Characteristics of patients with normal and elevated total serum IgE levels (shown are mean values). Analysis is based on 2280 patients.

	IgE < 100 IU/l	IgE > 100 IU/l	p-value
**Gender: male**	848 (54.08%)	490 (68.82%)	< 0.001
**Gender: female**	720 (45.92%)	222 (31.18%)	
**Weight (kg)**	78.81	79.54	NS
**Height (kg)**	169.92	171.83	< 0.001
**BMI**	27.18	26.85	NS
**Packyears**	47.87	49.72	NS
**Age (years)**	64.74	64.95	NS
**FEV_1 (% predicted)**	0.60	0.60	NS
**ITGV (% predicted)**	145.28	140.85	0.009
**DLCO (% predicted)**	54.35	57.43	NS
**SGRQ**	42.41	43.13	NS
**CAT score**	17.91	18.33	NS

Table S2B

Characteristics of patients with normal and elevated allergen-specific serum IgE levels (SX1)(shown are mean values). Analysis is based on 2280 patients.

	SX1 < 0.35 IU/l	SX1 > 0.35 IU/l	p-value
**Gender: male**	978 (57.56%)	350 (62.06%)	< 0.001
**Gender: female**	721 (42.44%)	214 (37.94%)	
**Weight (kg)**	78.84	79.69	NS
**Height (kg)**	170.24	171.39	< 0.001
**BMI**	27.09	27.04	NS
**Packyears**	48.92	47.23	NS
**Age (years)**	64.97	64.23	NS
**FEV_1 (% predicted)**	0.59	0.64	NS
**ITGV (% predicted)**	145.64	138.44	0.009
**DLCO (% predicted)**	53.74	60.03	NS
**SGRQ**	43.05	41.43	NS
**CAT score**	18.15	17.70	NS

Table S3

Blood eosinophil data (visit 1) were available from 594 patients. In this subgroup, there was a weak correlation between eosinophil percentages ($r = 0.09$, $p < 0.05$) and concentrations ($r = 0.08$, $p < 0.05$) and total IgE. There was no correlation between SX1 levels and eosinophils (data not shown). Patients with elevated total IgE or allergen-specific IgE (SX1) displayed slightly higher blood eosinophil percentages and cell counts, as compared to patients with normal IgE values:

	**Normal IgE (< 100 IU/ml)**				**Elevated IgE (≥ 100 IU/ml)**			
**Eosinophils**	Median	Interquartile Range	Mean	Standard deviation	Median	Interquartile Range	Mean	Standard deviation
cells/µl blood	160	140	180	150	160	180	220	180
% of all leukocytes	2.00	2.00	2.26	1.85	2.00	2.33	2.78	2.33
	**Normal SX1 (<0.35 IU/ml)**				**Elevated SX1 (≥ 0.35 IU/ml)**			
cells/µl blood	140	150	180	160	160	150	210	180
% of all leukocytes	2.00	2.00	2.35	1.96	2.00	1.90	2.65	2.24

Shown are blood eosinophil concentrations (cells / µl blood) and percentages (% of all blood leukocytes) of patients with normal and elevated total IgE and allergen-specific serum IgE levels (SX1). All differences were statistically significant ($p < 0.05$).

Table S4A

Logistic regression analyses for the association between tertiles of total serum IgE at baseline and risk of declining FEV_1 (group C) during follow-up. Tertiles of total IgE: tertile 1: ≤ 24.2 IU/ml, tertile 2: 24.3 - 91.5 IU/ml, tertile 3: > 91.5 IU/ml. The minimal sufficient adjustment sets for estimating the total effect of total IgE on declining FEV_1 comprises age and gender (according to DAG diagram analyses, www.dagitty.net). Therefore, we calculated logistic regression models adjusting for age and gender (model 1) and additional models with more potential confounders (model 2: adjusted for age, gender, BMI, initial FEV_1 % predicted, smoking status; model 3: adjusted for age, gender, BMI, initial FEV_1 % predicted, smoking status, history of asthma, history of allergy).

Model	Tertile of IgE	OR	95 % CI	P
**Crude**	1	Reference		
	2	1.01	0.80-1.29	0.910
	3	1.57	1.09-2.26	0.015
**Model 1**	1	Reference		
	2	1.01	0.79-1.29	0.936
	3	1.58	1.09-2.27	0.015
**Model 2**	1	Reference		
	2	1.01	0.80-1.29	0.912
	3	1.59	1.10-2.30	0.013
**Model 3**	1	Reference		
	2	1.00	0.78-1.28	0.999
	3	1.62	1.12-2.34	0.011

Model 1: Adjusted for age, gender.
Model 2: Adjusted for age, gender, BMI, initial FEV_1 % predicted, smoking status.
Model 3: Adjusted for age, gender, BMI, initial FEV_1 % predicted, smoking status, history of asthma, history of allergy.

Table S4B

Logistic regression analyses for the association between tertiles of allergen-specific IgE (SX1) at baseline and risk of declining FEV_1 (group C) during follow-up. Tertiles of SX-1: tertile 1: ≤ 0.08 IU/ml, tertile 2: 0.09 - 0.19 IU/ml, tertile 3: > 0.19 IU/ml. The minimal sufficient adjustment sets for estimating the total effect of SX1 on declining FEV_1 comprises age and gender (according to DAG diagram analyses, www.dagitty.net). Therefore, we calculated logistic regression models adjusting for age and gender (model 1) and additional models with more potential confounders (model 2: adjusted for age, gender, BMI, initial FEV_1 % predicted, smoking status; model 3: adjusted for age, gender, BMI, initial FEV_1 % predicted, smoking status, history of asthma, history of allergy).

Model	Tertile of SX1	OR	95% CI	P
**Crude**	1	Reference		
	2	1.08	0.78-1.49	0.647
	3	2.03	1.27-3.23	0.003
**Model 1**	1	Reference		
	2	1.07	0.78-1.48	0.670
	3	1.97	1.23-3.15	0.005
**Model 2**	1	Reference		
	2	1.11	0.80-1.55	0.522
	3	2.10	1.30-3.38	0.002
**Model 3**	1	Reference		
	2	1.13	0.81-1.58	0.484
	3	2.15	1.32-3.51	0.002

Model 1: Adjusted for age, gender.
Model 2: Adjusted for age, gender, BMI, initial FEV_1%, smoking status.
Model 3: Adjusted for age, gender, BMI, initial FEV_1%, smoking status, history of asthma, history of allergy.

Fig. S1

Correlation as measured using Spearman's correlation coefficient (r) between total IgE levels in serum (x-axis) and allergen-specific IgE in serum (y-axis, logarithmic scale) as measured using the SX assay, in the total group of 2280 patients. *** = $p < 0.001$.

Fig. S2

Total and allergen-specific IgE (SX) in serum according to spirometric GOLD grades 1-4. The percentage of patients with ≥ 100 IU/l total IgE (upper panel) or ≥ 0.35 IU/l allergen-specific IgE (lower panel) are shown in yellow (with specific percentages), for males and females with COPD. There was a weak correlation between allergen-specific IgE (SX1) and FEV1 (% predicted), in males ($r = 0.09$, $p = 0.001$) and females ($r = 0.07$, $p = 0.033$). Accordingly, the percentage of patients with elevated allergen-specific IgE (SX1) levels decreased from spirometric GOLD grades 1 to 4. There were weak correlations between allergen-specific IgE (SX1) and DLCO (males: $r = 0.11$, $p < 0.001$; females: $r = 0.12$, $p < 0.001$), RV (males: $r = - 0.06$, $p = 0.039$; females: $r = - 0.11$, $p = 0.001$) and ITGV (males: $r = - 0.06$, $p = 0.039$; females: $r = - 0.09$, $p = 0.007$).

Fig. S3

Restricted cubic splines to visualize the non-linear association between total IgE and the exacerbation history. Three knots of total or allergen-specific IgE were placed corresponding to the 10th, 50th, and 90th percentile of the IgE levels, respectively. Increasing total IgE levels were associated with a higher risk of exacerbations in men ($p = 0.004$), but not in women ($p = 0.135$) with COPD.

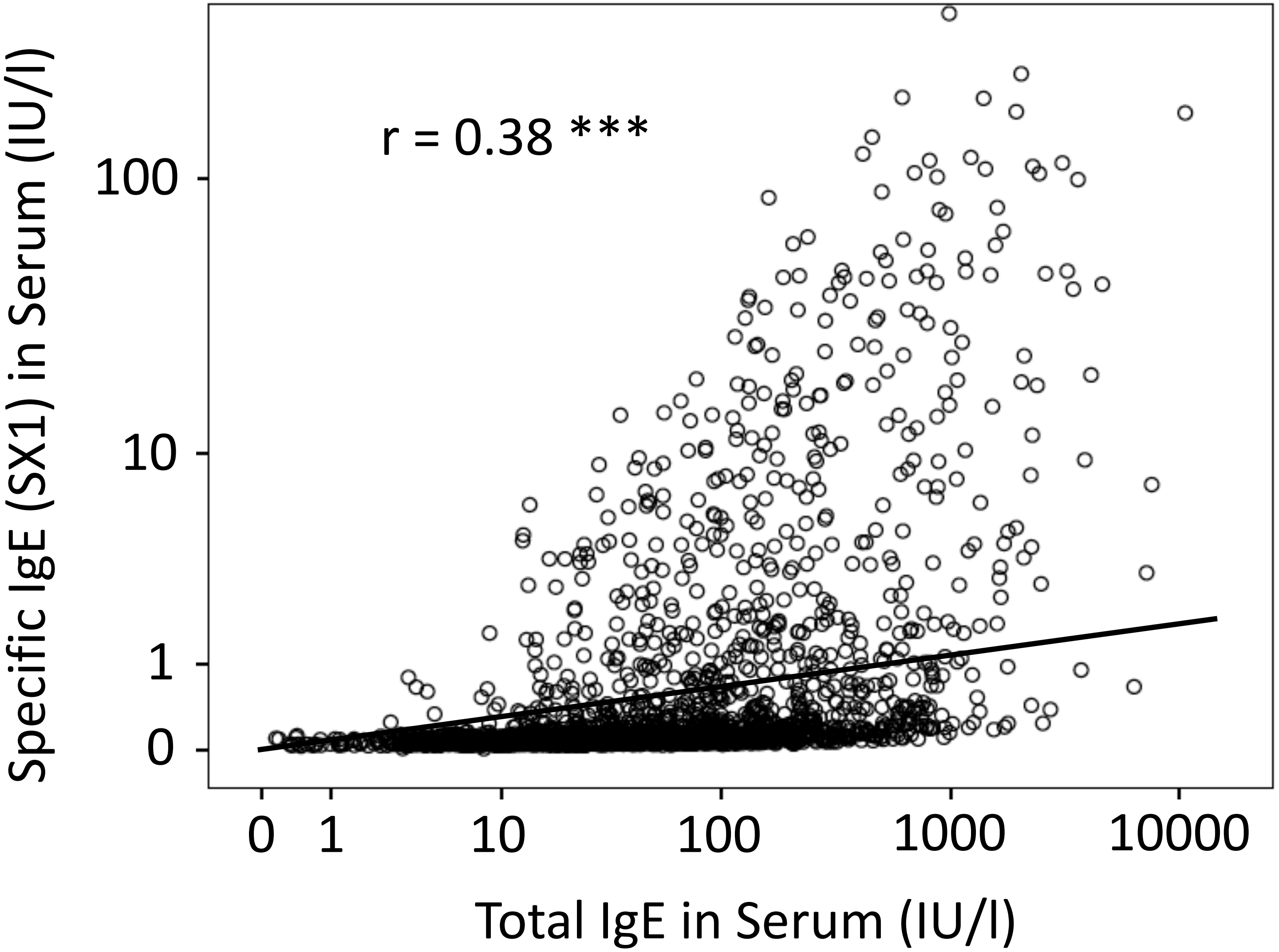
r = 0.38 ***
Specific IgE (SX1) in Serum (IU/l)
100
10
1
0
0
1
10
100
1000
10000
Total IgE in Serum (IU/l)

Fig. S1

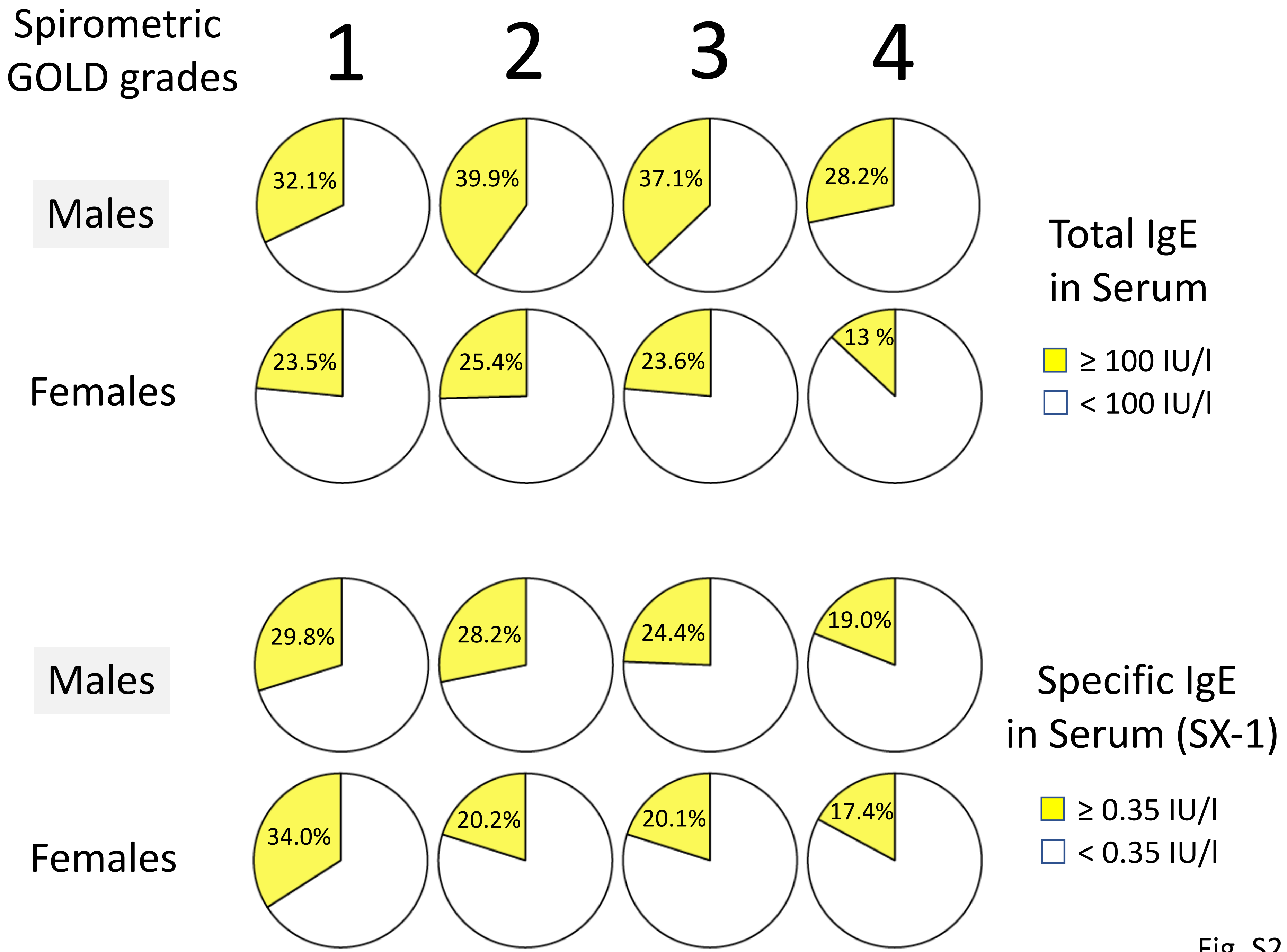
Spirometric GOLD grades
1
2
3
4
Males
32.1%
39.9%
37.1%
28.2%
Females
23.5%
25.4%
23.6%
13 %
Total IgE in Serum
≥ 100 IU/l
< 100 IU/l
Males
29.8%
28.2%
24.4%
19.0%
Females
34.0%
20.2%
20.1%
17.4%
Specific IgE in Serum (SX-1)
≥ 0.35 IU/l
< 0.35 IU/l

Fig. S2

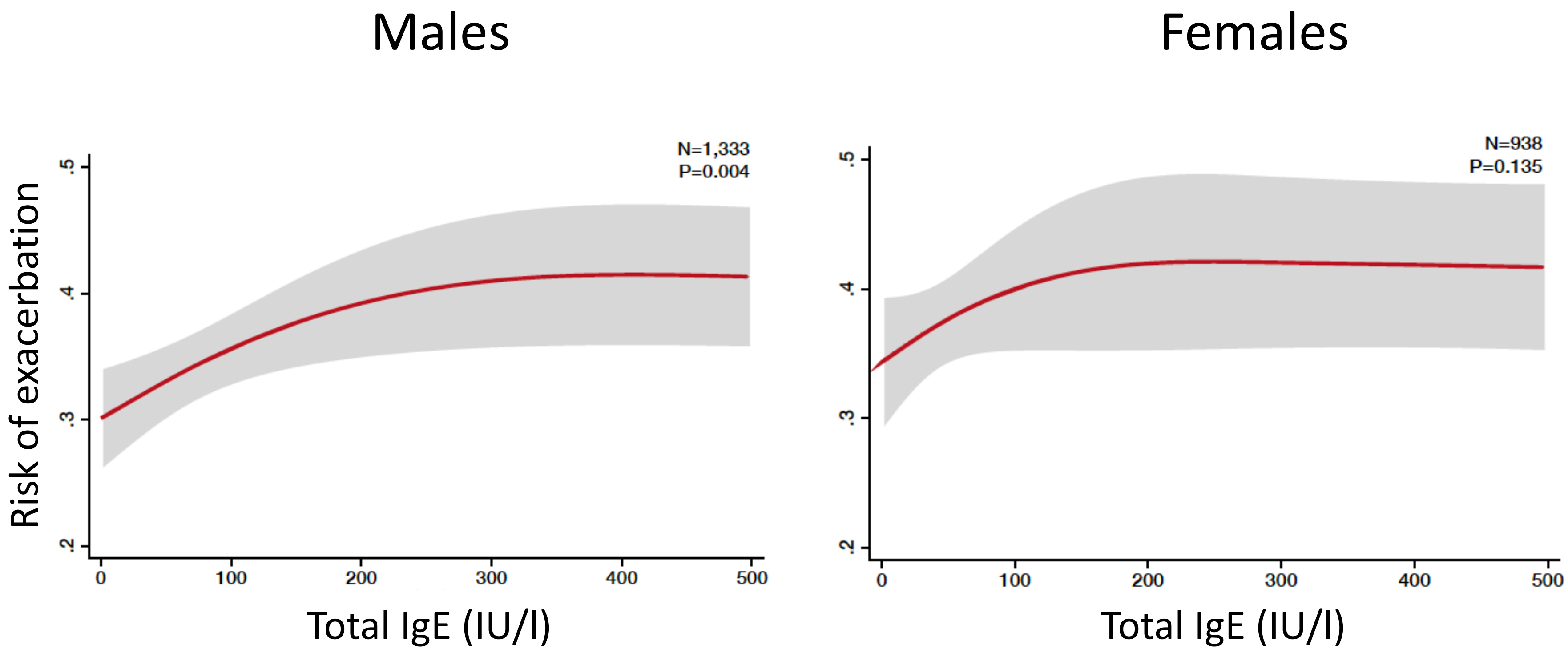

Males
Females
N=1,333
P=0.004
N=938
P=0.135
Risk of exacerbation
.2
.3
.4
.5
0
100
200
300
400
500
Total IgE (IU/l)
Total IgE (IU/l)

Fig. S3
